# Distinct host-immune response toward species related intracellular mycobacterial killing: A transcriptomic study

**DOI:** 10.1080/21505594.2020.1726561

**Published:** 2020-02-13

**Authors:** Abhilasha Madhvi, Hridesh Mishra, Novel N. Chegou, Gerard Tromp, Carel J. Van Heerden, R. D. Pietersen, Gina Leisching, Bienyameen Baker

**Affiliations:** a NRF-DST Centre of Excellence for Biomedical Tuberculosis Research; South African Medical Research Council Centre for Tuberculosis Research; Division of Molecular Biology and Human Genetics, Faculty of Medicine and Health Sciences, Stellenbosch University, Cape Town, South Africa; b South African Tuberculosis Bioinformatics Initiative (SATBBI), Faculty of Medicine and Health Sciences, Stellenbosch University, Cape Town, South Africa; c Centre for Bioinformatics and Computational Biology, Stellenbosch University, Cape Town, South Africa; d DNA Sequencing Unit, Central Analytical Facility (CAF), Stellenbosch University, Stellenbosch, South Africa

**Keywords:** Mycobacteria, pathogenic, nonpathogenic, host immune response, amplicon-based RNA sequencing, detergent-free media

## Abstract

The comparison of the host immune response when challenged with pathogenic and nonpathogenic species of mycobacteria can provide answers to the unresolved question of how pathogens subvert or inhibit an effective response. We infected human monocyte derived macrophages (hMDMs) with different species of mycobacteria, in increasing order of pathogenicity, i.e. *M. smegmatis, M. bovis* BCG, and *M. tuberculosis* R179 that had been cultured in the absence of detergents. RNA was isolated post-infection and transcriptomic analysis using amplicons (Ampliseq) revealed 274 differentially expressed genes (DEGs) across three species, out of which we selected 19 DEGs for further validation. We used qRT-PCR to confirm the differential expression of 19 DEGs. We studied biological network through Ingenuity Pathway Analysis® (IPA) which revealed up-regulated pathways of the interferon and interleukin family related to the killing of *M. smegmatis*. Apart from interferon and interleukin family, we found one up-regulated (*EIF2AK2)* and two down-regulated (*MT1A* and *TRIB3*) genes as unique potential targets found by Ampliseq and qRT-PCR which may be involved in the intracellular mycobacterial killing. The roles of these genes have not previously been described in tuberculosis. Multiplex ELISA of culture supernatants showed increased host immune response toward *M. smegmatis* as compared to *M. bovis* BCG and *M.tb* R179. These results enhance our understanding of host immune response against *M.tb* infection.

## Introduction

According to the World Health Organization (WHO) global tuberculosis (TB) report in 2018, TB is the number one infectious killer globally [1]. Different species of mycobacteria have been shown to elicit different immune responses [2]. Pathogenic mycobacteria are known to survive inside the host by arresting phagosome maturation [3–5]. The pathogen survives inside the optimal environment of the phagosome and the mechanism responsible for its growth and survival inside the host is still unknown [6–8].

The enigma of the host response to effectively counter nonpathogenic bacteria but being unable to destroy pathogenic bacteria may be deciphered by comparing the host immune response toward these pathogenic and nonpathogenic infections. One of the most widely used approaches for evaluating the host response to *M. tuberculosis (M. tb)* infection is transcriptome analysis using RNA sequencing [9 10,]. In the present study, we used an AmpliSeq^TM^, which is reported to be cost-effective, highly sensitive and reproducible for the detection of differentially expressed transcripts [11]. We also used Luminex technology as a multiplex immunoassay platform to study the levels of cytokines post-infection. Previous studies have also used multiplex ELISA for comparing the host immune response to infection at the protein level [12 13,]. The present study is a follow-up to our earlier work studying the initial host response to hyper-virulent *M. tb* infection in murine bone marrow-derived macrophages (BMDMs) [9].

The way in which the host cell responds to pathogenic (e.g. R179) and nonpathogenic mycobacteria (*M.smegmatis*) is believed to be largely different due to genomic and biochemical variability of mycobacteria [14]. In the present study, we used R179 *M. tb*, a known multi drug-resistant clinical isolate (Beijing genotype strain R220) prevalent in the Western Cape of South Africa [15]. We also used avirulent sub-strain of *M bovis* BCG (Tokyo 172) a mycobacterial species known for causing disease in both animals and humans [16]. We used *M. smegmatis* as nonpathogenic mycobacterial species which is known to be killed within 48 h of *in vitro* infection [17 18,]. The host can control *M. smegmatis* infection, although the factors behind this is understudied. We hypothesized that identifying these factors can help in developing interventions against pathogenic mycobacterial infection as well. Therefore, we compared the *in-vitro* host immune response from hMDMs toward mycobacteria of different pathogenicities using Ampliseq, qRT-PCR and multiplex ELISA, to elucidate the potential factors involved in countering the nonpathogenic mycobacteria (*M. smegmatis*).

## Materials and methods

### Enrollment of study participants

**Figure 1.**
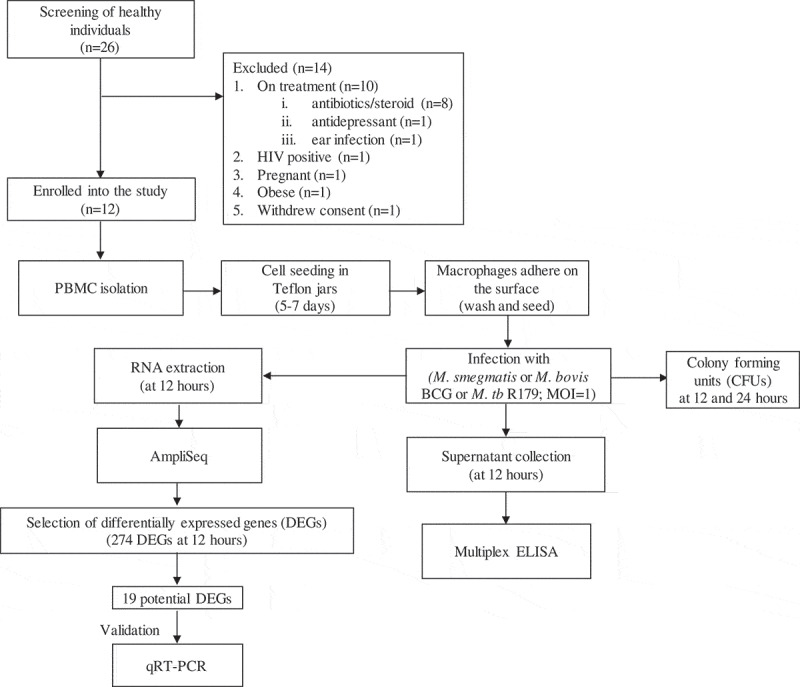
Study outline. Abbreviations: CFUs = colony-forming units, ELISA = enzyme-linked immunosorbent assay, PBMCs = peripheral blood mononuclear cells, DEGs = differentially expressed genes, MOI = multiplicity of infection, qRT-PCR = quantitative reverse transcriptase-polymerase chain reaction.

Healthy participants were screened for any symptoms of TB. Participants with cavities on chest X-ray, night sweats, recent unexplained weight loss, persistent cough, and fever were excluded. Additional exclusion criteria included: use of any medication, major surgery in the recent past, pregnancy, anemia, and insomnia. Additionally, we aimed to select equal representation of gender and ethnicity. We screened 26 individuals, out of which 14 were excluded (Figure 1). Twelve healthy participants (6 males and 6 females) with no history of TB were enrolled (Table 1).

Ethical approval for the study was obtained from the Health Research Ethics Committee (HREC), Stellenbosch University, Tygerberg campus, Cape Town (HREC Reference #S17/10/211).

### Cell isolation and culture medium

Blood (80 ml) was collected into Heparin Vacutainer (Lasec, South Africa, Cat No. VGRV455051R) by phlebotomy. The buffy coat was obtained by centrifugation and macrophage-like cells were isolated under culture conditions that allow differentiation of monocytes into macrophages, i.e. by culturing in Teflon-coated culture flasks using RPMI-1640 medium supplemented with 20% heparinized plasma and incubated at 37^°^C and 5% CO_2_ for 5 days.

After 5 days of culture, the cells were washed with phosphate buffer saline (PBS) and incubated in 12-well culture plates for 2 h at 37°C in a 5% CO_2_ incubator for adherence of hMDMs (otherwise lymphocytes will be difficult to remove) [19]. For infection experiments, human macrophage cells were seeded in 12-well plates with 0.7 × 10^6^ cells per well.

### Infection with mycobacteria

Pathogenic (R179), and nonpathogenic (*M. smegmatis* and *M. bovis* BCG) species of mycobacteria were used for infection. All three mycobacterial species were cultured in detergent-free media for infection. Mycobacteria were cultured in 7H9 media (Sigma Aldrich, USA) supplemented with 10% Oleic Albumin Dextrose Catalase (OADC) (Sigma Aldrich, USA) and 0.5% glycerol without Tween-80. We avoided use of Tween, since Tween is known to affect the rate at which macrophages ingest *M. tb* and form phagosomes as well as the subsequent immune response to *M. tb* [20].

A syringe-settle-filtrate (SSF) method was performed in order to disaggregate the detergent-free grown mycobacterial species, hence preparing the infecting doses equivalent for each strain. Stock cultures of mycobacteria stored at −80°C were thawed and disaggregated by pipetting 10 times with a 1-ml tip. Subsequently, the solution was passed through a 25-gauge needle 20 times [21] and any remaining aggregate was allowed to settle for up to 1 min [22]. The settling time was different for each species (*M.smegmatis *= 30 s, BCG = 1 min, R179 = 1 min). The top 750 µl of the solution was collected and added to 4.25 ml of RPMI-glutamine media. This 5 ml bacterial suspension was then immediately filtered through 5.0 µm pore size filter (Merck Millipore, Germany) to which 10% human serum (Celtic, Cat No. S4190) was added as a final concentration. This method was performed in our laboratory earlier to determine the effect of culturing the mycobacteria in the absence of a detergent as compared to its presence [20].

hMDMs were infected separately with each mycobacterial strain at multiplicity of infection (MOI) = 1. After 4 h of infection, cells were washed three times with PBS to remove any extracellular mycobacteria. Cells were then incubated for another 8 h in 5% CO_2_ incubator at 37°C. Uninfected hMDMs served as a control. Cells were then plated for colony-forming units (CFUs) and processed for RNA extraction. Culture supernatants were collected for measurement of cytokine levels using the Luminex platform (Biocom, USA, Cat. No. PPX-12).

### Determination of bacterial uptake

CFUs were determined in hMDMs infected with *M. smegmatis, M. bovis* BCG and R179 mycobacterial species. Infected cells were lysed using 0.1% Triton X-100 (Sigma Aldrich, Cat No. T8787). Bacterial uptake was determined by serial dilution (10^−1^ – 10^−4^) and plating out of mycobacteria onto 7H11 agar plates. Mycobacterial uptake was measured at 4 h and the mycobacteria survival within the infected cells was monitored at 12- and 24-h post infection. The percent uptake was calculated by dividing the number of bacteria taken up per milliliter by number of macrophages in each well.

### Cytotoxicity analysis

Cell cytotoxicity was tested with Roche WST-1 Cell Cytotoxicity Reagent (Roche, USA) in 1:10 dilution of WST-1 reagent to RPMI complete media. Since the hMDMs do not proliferate in a 4-day culture, cells at different time points (12- and 24-h post infection) were processed here. Cells were incubated for 1 h at 37° C in a 5% CO_2_ incubator. Absorbance was measured at two different wavelengths for correction (450 and 630 nm).

### RNA extraction

Total RNA from human macrophages was extracted using a kit (RNeasy Plus Mini Kit) per the manufacturer’s instructions. The extraction was performed immediately following the 12-h infection period. Genomic DNA was removed by column filtration “gDNA eliminator” column included in the kit. For each experiment, RNA quantity and quality was assessed using an Agilent 2100 Bioanalyzer. Only RNA samples with an RNA integrity number (RIN) ≥ 9 were used for amplicon-based RNA sequencing and qRT-PCR experiments. Total RNA was extracted and frozen immediately at −80°C until needed.

### Amplicon-based RNA sequencing

Transcriptome sequencing was performed at Central Analytical Facility (CAF) at Stellenbosch University, South Africa using the Ion AmpliSeq™ Transcriptome Human Gene Expression Kit (Thermo Fisher Scientific) according to the manufacturer’s protocol (protocol MAN0010742 Rv B.0). Total RNA (100 ng) was reverse-transcribed using the SuperScript® VILO™ cDNA Kit to generate cDNA. The 20.802 RefSeq genes were amplified for 10 reaction cycles using the Human Gene Expression Core Panel. After partial digestion, barcode adaptors (from the IonCde Adapters Kit) were ligated and the libraries were purified with Agencourt ™ AMPure™ XP reagent and eluted in low TE buffer. The concentrations of the RNA library were determined using the Ion Library TaqMan Quantitation Kit according to the protocol (MAN0015802 Rev A.0) and all libraries were diluted to a target concentration of 60 pM. To avert potential bias during sequencing or emulsion PCR, the 84 libraries were mixed before emulsion PCR, enrichment and chip loading on the Ion CHEF using the Ion PI™ Hi‑Q™ Chef Reagents, Solutions and Supplies according to the protocol (MAN0010967 Rev B.0). To limit the effect of run-to-run variation, all libraries were pooled and sequenced on multiple Ion P1™ v3 Chips on the Ion Proton™ according to the manufacturer’s protocol (MAN0010967 RevB.0).

Sequence data were analyzed using Torrent Suite Version 5.4.0 Software Partek Flow. All the reads were mapped by the TMAP module (as implemented in Torrent Suite). These mapped reads were exported as binary alignment and mapping (BAM) files (.bam extension) into the Partek Flow Software tool where the various data files for each sample were combined to create one dataset for each sample. After alignment, the QC module of Partek Flow was used to visualize the average base quality score per-position and per-alignment mapping. These mapped reads were quantitated using a separate set of software (RefSeq transcripts) annotation for quantiﬁcation using the Partek E/M method. Differential expression analyses was performed using “edgeR” version 3.4.1. Statistical analyses were carried out by the Division of Statistics at Stellenbosch University. We used edgeR to model a negative binomial distribution of count data and estimated dispersion parameters. Subsequently, we determined differential expression using one-way repeated measures ANOVA using Limma [23]. We used Benjamini-Hochberg false discovery rate to correct for multiple testing [24].

The edgeR statistical method was used to select DEGs. The DEG list was filtered using a combination of stringent gene filtering measures which included log counts per million (CPM), FDR <0.001 and fold changes (>1.5).

### qRT-PCR

Good quality RNA (RIN>9, 0.8 µg) was converted to cDNA using Quantitect® Reverse Transcription Kit. To ensure the removal of genomic DNA, “gDNA wipe-out buffer” was used. qRT-PCR ampliﬁcation was run on a LightCycler® 96 system (Roche, Germany). LightCycler® 480 SYBR Green I Master and QuantiTect® primer assays were used.

Hs-GAPDH and Hs-UBC were selected as reference genes conferring to stable expression levels from amplicon-based RNA sequencing data and validated through qRT-PCR. The ampliﬁcation process involved 45 cycles of 95°C for 10 s followed by 60°C for 10 s and ﬁnally 72°C for 10 s. Gene expression fold-changes were computed for pathogenic infected and nonpathogenic infected macrophages using calibrated, normalized relative quantities using the equation N = N_0_ × 2^Cp^. All qRT-PCRs were performed on RNA extracted from three separate experiments. All biological replicates, a positive control and a non-reverse transcription control were run in triplicate (along with calibrator) as per the MIQE Guidelines [25].

### Ingenuity Pathway Analysis (IPA®)

IPA® was performed using IPA Gene View Software (version 01–13). Statistical measures reflecting dataset genes interacting with each other (p-value) and activation (z-score) were based on the known direction of up- and down-regulated genes. For detailed pathway evaluation differentially expressed genes (DEGs) with fold changes >1.5 and adjusted p-value ≤ 0.05 were used in the analysis. These data was also used to generate lists of top Canonical Pathways, top upstream regulators and top regulator effect networks in hMDMs after infection with mycobacteria with respect to potential DEGs which were selected based on the stringent gene filtering measures.

### Multiplex ELISA

A panel of 7 cytokines was selected from the list of 19 DEGs. This panel included IDO-1, five interleukins (IL-1β, IL-6, IL-8, IL-12-β [IL-12p40, IL-12p70] and IL-23) and type-II interferon (IFN-γ) which was designed for studying their host response upon infection with mycobacteria at different time points using the Luminex platform. Cytokine concentrations were evaluated using on the Bioplex platform (BioRad Laboratories, Hercules, CA, USA) using Procarta Luminex kits – ThermoFisher (Cat No. PPX-12) according to the manufacturer’s protocol.

The coefficient of variation for duplicate runs was <20% for all samples (range, 5.2–19.6%) and the concentrations of all analytes in the quality control reagents were within their expected ranges. The standard curve for all samples ranged from 3.6 to 10,000 pg/ml. Bioplex Manager Software version 1 (BoiRad, south Africa) was used for analysis of median fluorescent intensities.

## Statistical analysis

The qRT-PCR data were analyzed using Light Cycler 96 SW 1.1 Software and Graph-pad Prism Version 7. Relative Expression which measures target transcript in a treatment group to that of untreated group was measured through the software in response to the calibrator and non-transcription control. The significance of the effect of strain was determined by one-way ANOVA using GraphPad Prism (Graph-Pad Software Version 7, San Diego California, USA) and Tukey’s honest significant difference test to correct for multiple testing.

The reads were quality trimmed on the 3ʹ end using a 30-bp sliding window. When the average base quality value was below 16, the last 15 bases were trimmed, and the evaluation was performed for the next window of 30 bases. The qRT-PCR data in the study is presented as mean ± SD from 12 healthy participants.

Cytotoxicity graphs and CFUs were plotted with an average of the technical triplicates leading to the mean of all the biological replicates. Statistical analysis was performed through Graph-pad Prism V7 software where the percentage of every expressing cell was generated, and p-value was calculated using two-way ANOVA with Tukey’s correction. Luminex data were analyzed by two-way ANOVA with Tukey’s correction using Graph-Pad Prism.

## Results and discussion

The pathogenicity of different species of mycobacteria can be attributed, in part, to components of the bacterial cell wall, genome and metabolome that differ between species [26]. It is challenging to characterize host response-related factors, especially those that can control, or alter, pathogenicity of the mycobacteria. Recent publications used Ampliseq and RNAseq to compare the host response from alveolar macrophages and hMDMs against H37Rv, and found key genes (*TREM1* and *IL10*) involved in pathogenesis of TB [11 27,]. The biological factors that contribute to pathogenicity in mycobacteria remain poorly understood.

In the present study, we examined differential gene expression during the infection of hMDMs with pathogenic (*M. tb* R179; drug resistant Beijing genotype strain R220) and nonpathogenic (*M. smegmatis*) species of mycobacteria along with *M. bovis* BCG (BCG Tokyo 172 strain) [15 28 29,,]. Ampliseq analysis of RNA derived from hMDMs before and after infection across three mycobacterial species identified overlapped 274 DEGs at 12-h post infection. Out of which, 229 are upregulated and 45 are downregulated (Accession no. GSE 122,619). Out of 274 DEGs, we selected 19 DEGS of potentially higher importance after applying stringent filtering parameters using edgeR statistical method. A similar viability was observed at 12 and 24 h for hMDMs infected with all three species (Figure S3). We examined the process of intracellular killing at 12-h post *M. smegmatis* infection [17 18,].

Importantly, we used detergent-free mycobacterial cultures for the *in vitro* infection experiments, since detergents (Tween) are used to prevent cellular clumping of *M. tb*, but are known to alter bacterial uptake by macrophages and alter the subsequent innate immune response [30 31,].

**Figure 2.**
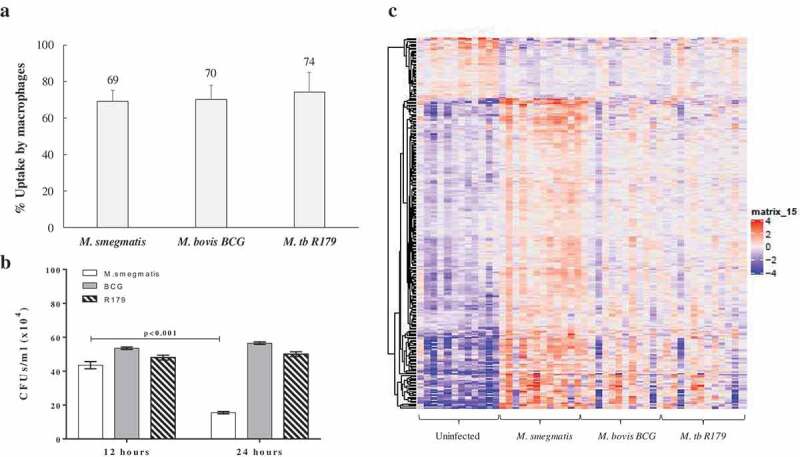
Uptake, CFUs and global transcriptome profile of hMDMs infected with mycobacteria. (a) Percent uptake of *M. smegmatis, M. bovis* BCG and *M. tb* R179 by hMDMs at 4 h, the percent uptake was calculated by dividing the number of bacteria taken up per milliliter by number of macrophages in each well., (b) CFUs of mycobacteria with multiplicity of infection (MOI) of 1 at 12- and 24-h post infection, and (c) heatmap of differentially expressed transcripts analyzed by AmpliSeq where hMDMs infected with *M. smegmatis, M. bovis* BCG or *M. tb* R179 were compared to uninfected hMDMs. The level of expression of each gene in each sample in comparison to the level of expression of uninfected sample is depicted with a color scale with red as maximum and blue as the minimum expression. *M. smegmatis* having the maximum red indicates stronger host response as compared to other two strains. Dendrogram indicates sample clustering. Abbreviations: BCG = Bacillus Calmette–Guerin, CFUs = colony-forming units, hMDMs = human monocyte derived macrophages, *M. tb = Mycobacterium tuberculosis*.

In the present study, the bacterial uptake by macrophages for *M. smegmatis, M. bovis* BCG and *R179* were confirmed to be similar (Figure 2(a)) and host-cell viability remained high (Figure S2). We confirmed intracellular killing of all three species using intracellular CFU counts at 12- and 24-h post infection. We observed a dramatic decrease in CFUs of *M. smegmatis* at 24 h compared to 12 h (p < 0.001), which was not observed for *M. bovis* BCG (p = 0.060) and *M. tb* R179 (p = 0.193) (Figure 2(b)). Also, CFUs of hMDMs infected with *M. bovis* BCG and *M. tb* R179 at 48-, 72- and 96-h post infection were found to significantly higher compared to 12-h post-infection (Figure S1). CFUs of hMDMs infected with *M. smegmatis* could not be determined beyond 24-h post-infection, as it is known to be killed within 48 h [17 18,]. This indicates that host can control *M. smegmatis* infection, although the reason behind this is understudied. We therefore present the host transcriptome at 12-h post-infection in response to pathogenic and nonpathogenic mycobacteria in an attempt to unravel the genes that are involved during this crucial time. In the present study, we have performed Ampliseq at only 12-h post-infection indicating higher expressions of DEGs in hMDMs infected with *M. smegmatis*, it is however highly likely that these genes continue to overexpress at 24-h post-infection to facilitate killing of mycobacteria. Results of amplicon-based RNA sequencing show the DEGs from hMDMs infected with three mycobacterial species compared to uninfected hMDMs (Figure 2(c)). Similar to a previous study [32], we found hMDMs infected with *M. smegmatis* had higher expression of DEGs when compared to *M. bovis* BCG and R179.

**Figure 3.**
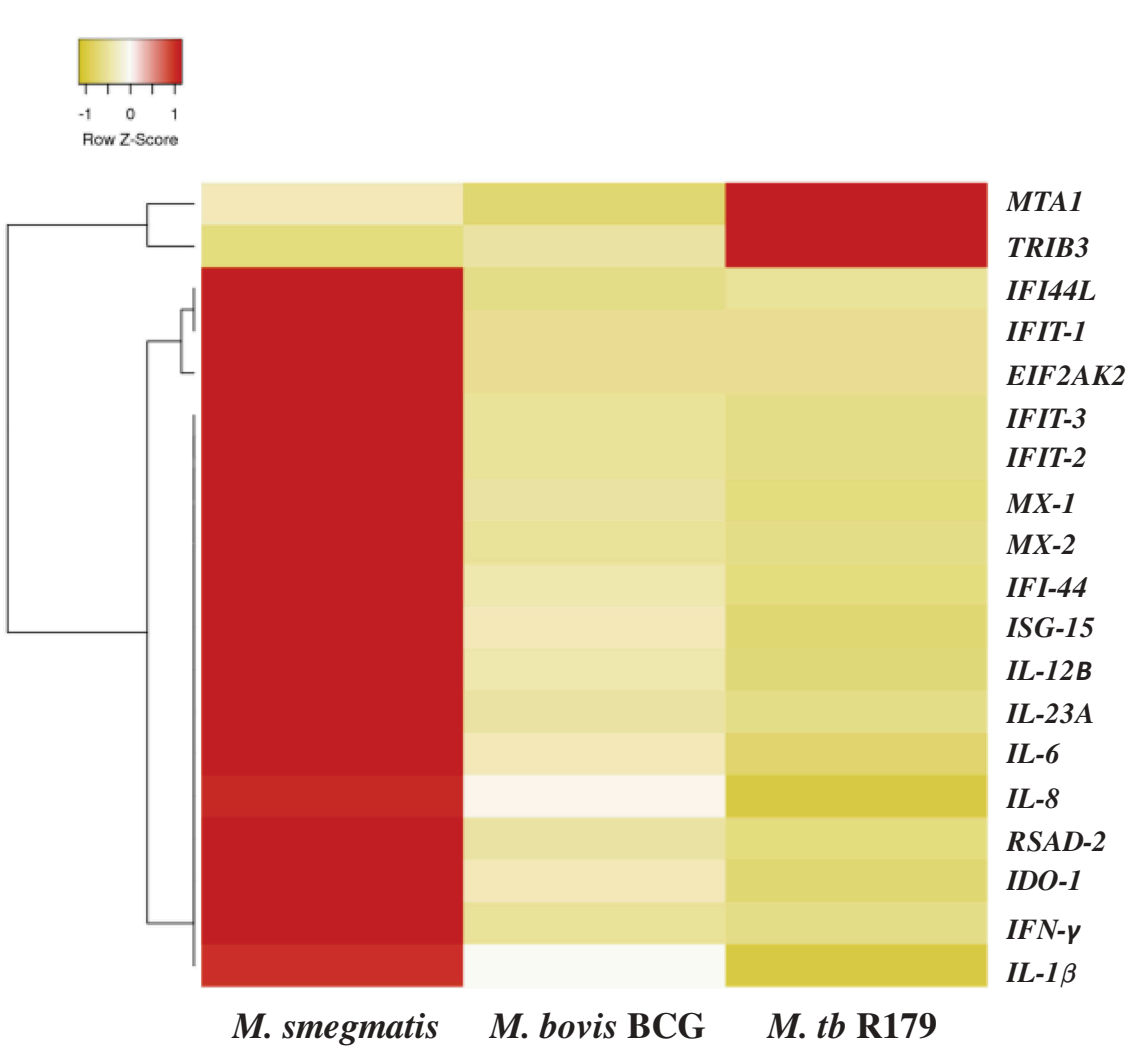
Differential expression of host gene transcripts in infected hMDMs. Heatmap of 19 differentially expressed transcripts with lowest false-discovery-rate analyzed by Ampliseq, hMDMs infected with *M. smegmatis, M. bovis* BCG or *M. tb* R179 were compared to uninfected hMDMs. The level of expression of each gene in each sample in comparison to the level of expression of uninfected sample is depicted with a color scale with red as minimum and white as the maximum expression. Dendrogram indicates sample clustering. Abbreviations: BCG = Bacillus Calmette–Guerin, hMDMs = human monocyte derived macrophages, *M. tb = Mycobacterium tuberculosis*

Ampliseq found 19 DEGs across the three mycobacterial species based on lowest false discovery rate (FDR<0.001). Out of these, 17 DEGs were upregulated and two downregulated upon *M. smegmatis* infection (Table 2). The heat map noticeably delineates 17 up-regulated and 2 down-regulated genes comprising largely interleukin (IL-1β, IL-6, IL-8, IL-12β and IL-23A) and interferon (IFN-γ, *IFI44, IFI44L, IFIT1, IFIT2, IFIT3, MX2* and *ISG15*) families (Figure 3).

**Table 1.** Demographic characteristics of study participants.

Variables	Study participants (n = 12)
Age (Years)	27
Median (IQR)	(26–28.5)
**Gender (%)**	
Female	6 (50%)
Male	6 (50%)
**BMI (kg/m^2^)** (Mean ± SD)	23.59 ± 2.32
**Ethnicity (%)**	
Black	6 (50%)
White	6 (50%)

**Abbreviations**: IQR = interquartile range, SD = standard deviation.

**Table 2.** Differentially expressed genes selected from AmpliSeq analysis of hMDMs 12-h post infection with pathogenic (*M. bovis* BCG, *M. tb* R179) and nonpathogenic (*M. smegmatis*) mycobacteria.

	*M. smegmatis*			*M. bovis* BCG			*M. tb* R179		
Gene name	Fold change	p value	FDR	Fold change	p value	FDR	Fold change	p value	FDR
*IFIT1*	6.3	3.36E-19	2.02E-15	1.6	1.16E-18	4.64E-15	1.8	6.09E-18	1.83E-14
*IFIT2*	4	2.27E-11	1.01E-08	1.6	2.5E-11	1.07E-08	1.8	3.76E-11	1.56E-08
*IFIT3*	6.9	2.53E-09	4.75E-07	2.8	2.95E-09	5.46E-07	2.5	3.87E-07	6.99E-07
*MX1*	4.6	6.05E-11	2.27E-08	1.6	6.51E-11	2.37E-08	1.7	6.7E-11	2.39E-08
*MX2*	3.4	1.85E-17	3.18E-14	1.9	1.45E-16	2.06E-13	1.8	1.54E-16	2.06E-12
*IFI44*	4	2.19E-08	3.29E-06	1.6	2.42E-08	3.59E-06	1.6	2.58E-08	3.78E-06
*IFI44L*	9	7.49E-11	2.58E-08	2.3	1.1E-10	3.69E-08	1.7	1.49E-10	4.7E-08
*ISG15*	3.5	7.23E-10	1.64E-07	1.7	8.71E-10	1.94E-07	1.9	1.08E-09	2.36E-07
*IL12B*	178.1	1.11E-13	8.38E-11	57.3	1.55E-13	1.1E-10	29.7	2.58E-13	1.73E-10
*IL23A*	9.1	6.86E-10	1.62E-07	3.2	7.03E-10	1.63E-07	1.7	5.92E-10	1.43E-07
*IL6*	12.4	5.64E-05	0.00029	6.5	5.89E-05	0.00023	4.6	6.07E-05	0.00023
*IL8*	8.7	2.45E-06	0.00021	7.2	2.46E-06	0.00021	5.9	2.83E-06	0.000238
*RSAD2*	7.9	5.62E-07	5.64E-05	3.1	6.75E-07	6.71E-05	2.6	6.8E-08	6.71E-05
*TRIB3*	−7.5	3.12E-07	0.00031	−7	4.4E-07	0.00041	−1.9	5.23E-07	0.00051
*MT1A*	−32	1.42E-05	0.00053	−41	1.42E-05	0.00041	−1.8	1.52E-05	0.00053
*IDO1*	12.6	1.79E-05	0.00015	6.6	1.8E-05	0.00011	5	1.81E-05	0.00014
*EIF2AK2*	2.3	4.11E-34	4.94E-30	1.6	8.47E-18	1.91E-14	1.6	9.51E-18	1.91E-14
*IFN-γ*	14.4	7.08E-07	6.93E-05	3.9	7.29E-07	7.08E-05	3.4	7.53E-07	7.25E-05

Abbreviations: BCG, Bacillus Calmette–Guerin; EIF2AK2, eukaryotic translation initiation factor 2 alpha kinase 2; FDR, false discovery rate; IDO, indoleamine 2,3-dioxygenase; hMDMs, human monocyte-derived macrophages; IFI, interferon-induced protein; IFIT, interferon-induced protein with tetratricopeptide; IFN, interferon gamma; IL, interleukin; ISG, interferon-stimulated gene; MTA, metastasis-associated protein; MX, interferon-induced GTP binding protein; RSAD, radical S-adenosyl methionine domain-containing protein 2; TRIB, trible homolog; UBC, polyubiquitin-C. Figure 3 is the pictorial heat-map of these results.

There are some mechanisms through which the macrophage host immune response controls the growth of *M. smegmatis*. A previous study showed that phagocytosis of *M. smegmatis* induces reactive oxygen species (ROS), resulting in the production of proinflammatory cytokines. ROS induced during phagocytosis is associated with the *M. smegmatis*-mediated endoplasmic reticulum (ER) stress response in macrophages [33]. Another study showed that *M. smegmatis* harboring unique multigenic PE_PGRS41 protein boosted the survival within macrophage accompanied with enhanced cytotoxic cell death through inhibiting autophagy and cell apoptosis [34].

**Figure 4.**
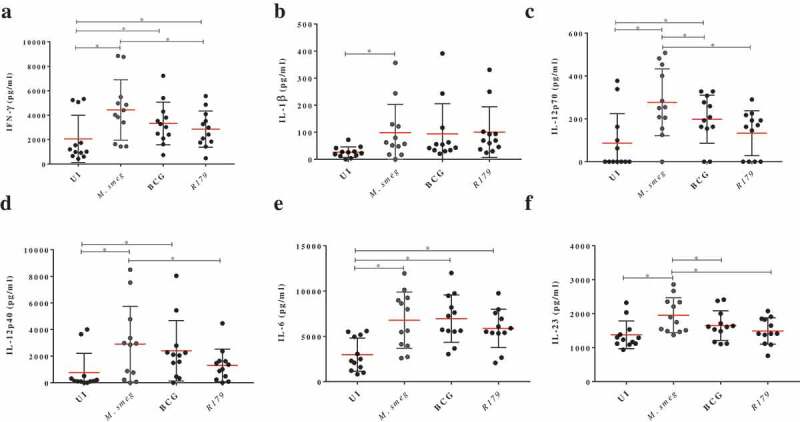
Cytokines levels after infection of hMDMs with *M. smegmatis, M. bovis* BCG or *M. tb* R179 compared to uninfected hMDMs measured through multiplex ELISA. (a) INF-γ, (b) IL-1β, (c) IL-12p40, (d) IL-12p70, (e) IL-6, and (f) IL-23. Detailed comparison of multiplex ELISA results are in Table S1. Abbreviations: BCG = Bacillus Calmette–Guerin, hMDMs = human monocyte derived macrophages, IDO-1 = indoleamine 2,3-dioxygenase-1, IFN = interferon, IL = interleukin, *M. tb = Mycobacterium tuberculosis*﻿.

**Figure 5.**
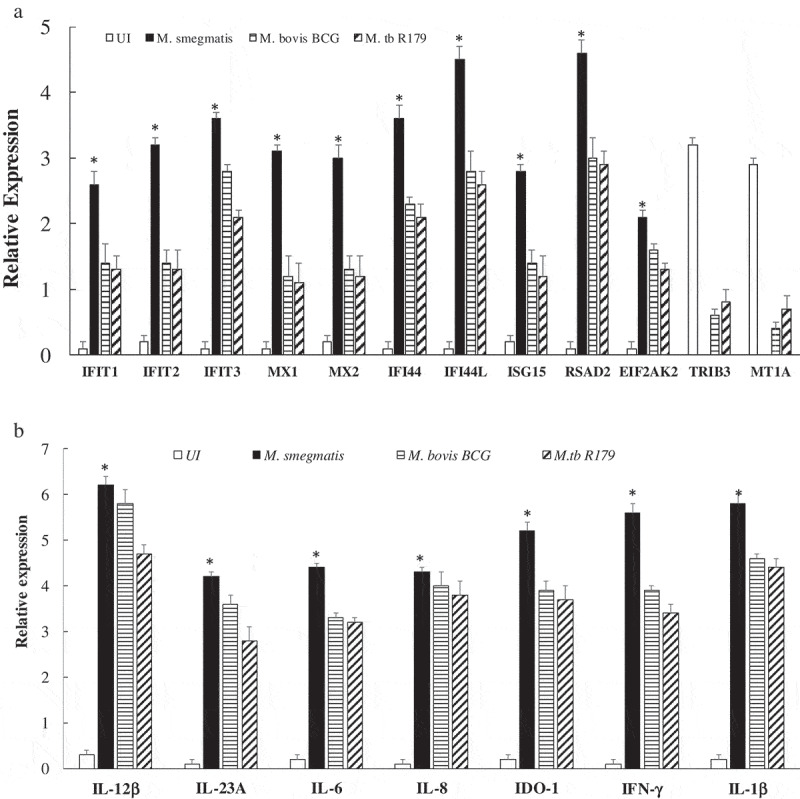
Expression analysis using qRT-PCR of twelve key transcriptomes (a) and seven key cytokines (b) in hMDMs of 12 healthy participants infected with *M. smegmatis, M. bovis* BCG or *M. tb* R179 as compared to uninfected hMDMs. (*) depicts significant (p < 0.05) expression. Abbreviations: BCG = Bacillus Calmette–Guerin, hMDMs = human monocyte-derived macrophages, *IFIT1 *= interferon-induced protein with tetracopeptide, *MX *= interferon-induced GTP binding protein, *IFI *= interferon-induced protein, *ISG *= interferon-stimulated gene, *RSAD *= radical s-adenosyl methionine domain-containing protein, *EIF2AK2 *= eukaryotic translation initiation factor 2 alpha kinase 2, *TRIB3 *= trible homolog, *MTA *= metastasis-associated protein.

In the present study, biological network analysis found primary up-regulated pathways of interferon and interleukins family involved in the killing of *M. smegmatis* (Figure S4). The increase or decrease in the downstream functional outcomes are caused by the activated or inhibited upstream regulators, hence termed as regulator effect network. For each regulator effect network, a consistency score was calculated, where higher scores are given to networks with consistent directions [35]. In the present study, top regulator effect networks of the 19 DEGs demonstrated an important role in cell activation (Figure S4), DNA binding and viral replication. With a higher consistency score predicting a unidirectional consistent gene finding directed us toward DNA activation and cellular binding (Tables 3–5). Infection with *M. tb* is known to affect DNA binding activity through IFN-γ activation [36]. It is noteworthy that the amplicon-based RNA-sequencing results were validated through qRT-PCR hence confirming the similar trend of up-regulatory and down-regulated genes. The cytokines profile of IFN-γ, IL-12p40, IL-12p70 and IL-23 as assessed through multiplex ELISA was found to be significantly higher (p < 0.001) in *M. smegmatis* infection as compared to *M. bovis* BCG or R179. The levels of IL-1β and IL-6 was found to be similar across all three mycobacterial species (Figure 4(a–f)). mRNA expression level of these 19 DEGs measured by qRT-PCR confirms the Ampliseq results (Figure 5(a,b)). Upon *M. smegmatis* infection as compared to *M. bovis* BCG and R179 multiplex ELISA of eight pro-inflammatory cytokines (of 19 DEGs) showed a similar trend as observed in amplicon-based RNA sequencing and qRT-PCR results (Table S1). This included IDO-1, IFN-γ and six interleukins [IL-1β, IL-6, IL-8, IL-12p40, IL-12p70 and IL-23]. Multiplex ELISA (Figure 4(a–f)) results found four key cytokines, i.e. IL-12p40, IL-12p70, IL-23 and IFN-γ with higher expression in hMDMs infected with *M. smegmatis* (p < 0.001) as compared to *M. bovis* BCG and *M. tb* R179.

**Table 4.** Top upstream regulators activated in hMDMs after infection with mycobacteria with respect to 19 potential DEGs.

Upstream regulator	p-Value of overlap	Predicted activation
IFNL1	4.33E-26	Activated
TLR3	5.86E-25	Activated
IFN-β	1.56E-23	Activated
TLR9	4.68E-23	Activated
TLR7	4.81E-23	Activated

Abbreviations: hMDMs, human monocyte derived macrophages; DEGs, differentially expressed genes; IFNL, interferon ligand; TLR, toll like receptor. The data were analyzed through the use of IPA (QIAGEN Inc., https://www.qiagenbioinformatics.com/products/ingenuitypathway-analysis).

**Table 3.** Top canonical pathways activated in hMDMs after infection with mycobacteria with respect to 19 potential DEGs.

Regulators	Disease and functions	Consistency score
APP, CHUK, CSF2, EIF2AK2, IFNAR, IL-10, IL1β, MYD88, REL	Activation of cell, binding of DNA	119.05
IFN, IFNA/IFNA13, IFNA4, JAK1/2	Replication of viral replicon	8.05
IRF3	Immune response of cells	4.899
IRF3	Infection of Mammalia	4.899

Abbreviations: APP, amyloid precursor protein; CHUK, helix-loop-helix ubiquitous kinase; CSF2, colony-stimulating factor 2; DEGs, differentially expressed genes; EF2AK2, eukaryotic translation initiation factor 2-alpha kinase 2; hMDMs, human monocyte derived macrophages; IFNAR, type I interferon (IFN) receptor; IL-10, interleukin-10; IL-1β, interleukin-β; MYD88, myeloid differentiation primary response 88; REL, proto-oncogene c-rel; IFN, interferon; JAK, Janus kinases; IRF, Interferon regulatory factor 1. The data were analyzed through the use of IPA (QIAGEN Inc., https://www.qiagenbioinformatics.com/products/ingenuitypathway-analysis).

**Table 5.** Top regulator effect networks in hMDMs after infection with mycobacteria with respect to 19 potential DEGs.

Pathway	p-Value	Overlap (%)
Interferon signaling	9.51E-08	11.1%
Cytokines mediated communication between immune cells	8.03E-08	7.4%
Pattern Recognition Receptors in recognition of bacteria and viruses	3.43E-07	2.9%
Hypercytokinemia/hyperchemokinemia in the Pathogenesis	4.87E-06	7%
Hematopoiesis from pluripotent stem cells	6.08E-05	6.2%

Abbreviations: hMDMs, human monocyte derived macrophages; DEGs, differentially expressed gene. The data were analyzed through the use of IPA (QIAGEN Inc., https://www.qiagenbioinformatics.com/products/ingenuitypathway-analysis).

IFN-γ is primarily responsible for activation of macrophages and bactericidal activity [37]. It plays a key role in apoptotic induction through a nitric-oxide dependent pathway in macrophages infected with mycobacteria [38 39,]. We observed an increase in IL-12p40 and IL-12p70, which are the key members of IL-12 family and are primarily secreted by antigen-presenting cells and macrophages [40 41,]. IL-12 is known to enhance the synthesis of IFN-γ during infection [42]. Further, IL-12 and IL-23 have been demonstrated to confer protective cellular responses and promote host survival against *M. tb* [43]. Table S1 provides a detailed comparison of multiplex ELISA results measured at 12-h post-infection.

Importantly, IDO-1, IL-1β and IL-8 expression (at the transcript level) was found to be increased in hMDMs infected with *M. smegmatis* compared to the other two species in amplicon-based RNA sequencing and qRT-PCR. We found no significant difference between protein levels of IDO-1, IL-1β or IL-8 across the three mycobacterial species compared to uninfected hMDMs (Figure S2).

Previous studies also demonstrated that *M. tb* up-regulates host IL-6 production to inhibit type I interferon [44]. Additionally, IL-8 has been shown to play an instrumental role in cell recruitment since IL-8 is the major chemokine responsible for recruiting neutrophils [45]. IDO-1 has been shown to be increased in a murine model of TB in previous studies [46].

Interestingly, in our exploration using amplicon-based RNA sequencing (and confirmed by qRT-PCR), we found metastasis-associated protein-1A (*MT1A*) and trible homolog (*TRIB3*) exhibiting significantly different expression levels in hMDMs infected with *M. smegmatis* compared to *M. bovis* BCG and *M. tb* R179 (Figures 3 and 5). *TRIB3* is a protein kinase that negatively regulate NF-kB and sensitize cells to TNF- and TRAIL-induced apoptosis [47]. Also, *TRIB 3* can negatively regulate the AKT 1 (serine/threonine-protein kinase B) altering cell survival [48]. Another DEG, *MT1A* gene is *a* member of the metallothionein family and act as anti-oxidants, protect against free radicals of hydroxyl, and help in detoxification of heavy metals [49 50,]. The third DEG, *EIF2AK2* is a dsRNA-dependent serine/threonine-protein kinase induced by IFN, which plays an important role in the innate immune response against viral infection and is found to be involved in the regulation of apoptosis, signal transduction, cell proliferation and differentiation. *EIF2AK2* inhibits viral replication by targeting alpha subunit of eukaryotic initiation factor 2 (EIF2S1) via phosphorylation, which impairs the recycling of EIF2S1 leading to inhibition of translation which subsequently results in cessation of cellular and viral protein synthesis [51]. Notably, the downregulated DEGs *TRIB-3* and *MT1A* were found to be similarly expressed by macrophages infected with *M. smegmatis* and *M. bovis* species, which were significantly lower compared to macrophages infected with pathogenic *M. tb* R179. Previous studies have observed an increased level of *TRIB3* in patients with colorectal cancer and its expression suggests poor overall survival [52]. Also, its expression is found to have poor prognosis in patients with non-small cell lung cancer and breast cancer [53 54,]. The *MT1A* gene was found to be linked to oxidative stress, carcinogenesis, and obesity [55–57]. Genes *TRIB3* and *MT1A* are understudied in TB and are downregulated in *M. smegmatis* as compared to other two species, knocking-down these genes in pathogenic *M. tb* infected cells could yield lower CFUs indicating a potential immune-therapeutic candidate.

*EIF2AK2* expression was found to be increased only in *M. smegmatis* infection when compared to the other two species. Previous gene profiling meta-analysis found *EIF2AK2* as a shared gene between TB and rheumatoid arthritis [58]. *EIF2AK2* has previously been studied in viral infection and found to block translation of viral mRNA and promote cellular apoptosis [59]. This upregulated gene is not well studied in TB and certainly warrants further investigations.

The limitations of the present study include: 1) we selected only three mycobacterial species (pathogenic and nonpathogenic) to study the host response. The future studies can increase number and types of species in order to study a broader response of the host toward the mycobacteria. 2) We studied only one time point for amplicon-based RNA-sequencing analysis. 3) We have not performed any experiments to determine the role of any of the 19 DEGs (including *EIF2AK2, MT1A*, and *TRIB3*) in intracellular mycobacterial killing/survival. We have planned functional gene knock-down (using siRNA) and gene knock-up (using vector-derived overexpression) experiments for follow-up study.

In conclusion, we found 19 differentially expressed genes against mycobacteria, in hMDMs infected with *M. smegmatis* as compared to *M. bovis* BCG and *M. tb* R179. Out of which three are unique (*EIF2AK2, MT1A*, and *TRIB3*) DEGs. These unique targets should be studied for their functional properties (using knock-down/knock-up approach) to access their role in the intracellular killing of pathogenic mycobacteria.

## Data Availability

AmpliSeq^TM^ data have been deposited in the NCBI Gene Expression Omnibus (GEO) database with experiment series accession number [GSE122619]. https://www.ncbi.nlm.nih.gov/geo/query/acc.cgi?acc=GSE122619
