# Supplementary material for: Distinct host-immune response toward species related intracellular mycobacterial killing: A transcriptomic study
Source: Virulence. 2020 Feb 13;11(1):170–82. doi: 10.1080/21505594.2020.1726561 (PMC7051142; doi:10.1080/21505594.2020.1726561)
Supplement: Supplemental Material [file kvir-11-01-1726561-s001.docx]

Supplement:

Figure legends

Figure S1: CFUs of mycobacteria with multiplicity of infection (MOI) of 1 measured at 12, 24, 48, 72 and9 96 hours post-infection.

Abbreviations: BCG=Bacillus Calmette-Guerin, CFUs=colony forming units, hMDMs= human monocyte derived macrophages, *M.tb=Mycobacterium tuberculosis*.

Figure S2: Levels of seven cytokines after infection of hMDMs with *M. smegmatis, M. bovis* BCG and *M. tb* R179 compared to uninfected hMDMs measured through multiplex ELISA, A) IDO-1, B) IL-8 levels were found to be similar in hMDMs infected across all three mycobacterial species.

Abbreviations: BCG=Bacillus Calmette-Guerin, hMDMs= human monocyte derived macrophages, IDO-1=indoleamine 2,3-dioxygenase-1, IL=interleukin, *M. tb=Mycobacterium tuberculosis*, UI=uninfected.

Figure S3: Host cell viability upon infection with mycobacterial strains was measured through Cytotoxicity at 12 and 24 hours post-infection. Data represents mean of twelve individual experiments with standard deviation. Since the hMDMs do not proliferate in a 4 day culture, hence cells at different time points (12, and 24 hours post-infection) were processed with dilution 1:10 of WST-1: complete RPMI.

Abbreviations: hMDMs, human monocyte derived macrophages; RPMI,  Roswell Park Memorial Institute; WST, water-soluble tetrazolium salt; BCG=Bacillus Calmette-Guerin.

Figure S4: Canonical pathways generated through Ingenuity Pathway analysis (IPA). a) Inter-related network of 19 DEGs where, white to red colour depicting increase in up-regulation of DEGs.

Abbreviations: DEGs, differentially expressed genes; *EIF2AK2*, Eukaryotic translation initiation factor 2 alpha kinase 2; FDR, False discovery rate; IDO, Indoleamine 2,3-dioxygenase; hMDMs, human monocyte derived macrophages; IFI, Interferon induce protein; IFIT, Interferon induced protein with tetratricopeptide; IFN, Interferon gamma; IL, Interleukin; ISG, Interferon stimulated gene; *MTA*, Metastasis-associated protein; MX, Interferon induced GTP binding protein; *RSAD,* Radical S-Adenosyl Methionine Domain-Containing protein 2; *TRIB*, Trible homolog; UBC, Polyubiquitin-C.

Figure S1:

Figure S2:

Figure S3:

Figure S4:

Table S1: Expression of early response cytokines (pg/ml) from hMDMs before and after infection with pathogenic (*M. bovis BCG, M. tb R179*) and non-pathogenic (*M. smegmatis*) mycobacteria measured at 12 hours using multiplex ELISA.

| **Cytokine** | **Uninfected** | ***M. smegmatis*** | ***M. bovis* BCG** | ***M. tb* R179** |
| --- | --- | --- | --- | --- |
| **IDO-1** | 1763.3 ± 753.2 | 1916.1 ± 741.5  0.954* | 1843.6 ± 725.3  0.999*  0.994^†^ | 1870.6 ± 666.7  0.983*  0.998^†^  0.999^‡^ |
| **IFN-γ** | 639.7 ± 228.7 | 5710 ± 1533.5  <**0.0001*** | 2238.2 ± 781.8  **0.0008***  <**0.0001**^†^ | 1856.3 ± 733.9  **0.014***  <**0.0001**^†^  0.754^‡^ |
| **IL1-β** | 20.2 ± 19.1 | 119.4 ± 112.1  **0.021*** | 93.8 ± 111.5  0.129*  0.862^†^ | 84.9 ± 76.1  0.776*  0.180^†^  0.577^‡^ |
| **IL-12p70** | 17.8 ± 26.4 | 313.4 ± 143.8  <**0.0001*** | 198.4 ± 112.3  **0.0001***  **0.020**^†^ | 41.4 ± 38.9  0.927*  <**0.0001**^†^  **0.001**^‡^ |
| **IL-12p40** | 155.9 ± 142.5 | 3520.1 ± 2536  **0.0002*** | 2402.6 ± 2271.9  **0.020***  0.439^†^ | 1302.7 ± 1228.4  0.416*  **0.022**^†^  0.453^‡^ |
| **IL-6** | 1845.6 ± 663.1 | 7269.3 ± 2559.8  <**0.0001*** | 6957.6 ± 2611.1  <**0.0001***  0.984^†^ | 5885.1 ± 2116.7  **0.000***  0.397^†^  0.612^‡^ |
| **IL-23** | 1267.7 ± 190.7 | 2068.3 ± 475.6  <**0.0001*** | 1649.7 ± 436.3  0.090*  0.053^†^ | 1492.5 ± 387.7  0.495*  **0.003**^†^  0.755^‡^ |
| **IL-8** | 9953.3 ± 4555.2 | 12342.8 ± 2534.1  0.446* | 12275.9 ± 4009.4  0.471*  0.999^†^ | 13677.8 ± 4203.4  0.104*  0.836^†^  0.815^‡^ |

^*^= Uninfected v/s test (*M.smegmatis*, *M. bovis* BCG*, M. tb* R179), ^†^ = *M.smegmatis* v/s *M. bovis* BCG*, M. tb* R179, ^‡^ = *M. bovis* BCG v/s *M. tb* R179. Data represented as mean ± standard deviation.

Abbreviations: BCG=Bacillus Calmette-Guerin, hMDMs=human monocyte derived macrophages, IDO=indoleamine 2,3-dioxygenase, IFN=interferon, IL=interleukin, *M. tb*=*Mycobacterium tuberculosis*
